# Supplementary material for: MetaRibo-Seq measures translation in microbiomes
Source: Nat Commun. 2020 Jun 29;11:3268. doi: 10.1038/s41467-020-17081-z (PMC7324362; doi:10.1038/s41467-020-17081-z)
Supplement: Supplementary file 10 — Supplementary Data 7 [file 41467_2020_17081_MOESM10_ESM.zip › File2/Confidence_VeryHigh_Taxonomy/232457_out.krona.html]

Javascript must be enabled to view this page.

members
magnitude
magnitudeUnassigned
count
unassigned
taxon
rank

232457\_out

1
15

SRS147346\_contig\_number\_contig-100\_1615.339022


SRS075959\_contig\_number\_19940
2
1
superkingdom
11

phylum
10
976

class
10
200643

10
order
171549

171551
7
family

836
7
genus

997829

SRS012983\_contig\_number\_15735SRS014404\_contig\_number\_6031SRS015215\_contig\_number\_11546SRS077855\_contig\_number\_12659SRS1055039\_contig\_number\_17118SRS144621\_contig\_number\_69604
6
species

species
1

SRS021528\_contig\_number\_32409
41976

1853231

SRS104975\_contig\_number\_5015
1
family

171552
family
2

1
genus
838

species
1

SRS020540\_contig\_number\_8560
1262929

1
species
1852368

SRS056896\_contig\_number\_20571

superkingdom
3
2759

3
kingdom
4751

451864
subkingdom
3

4890
phylum
2

subphylum
2
147538

class
2
147550

subclass
1
222543

order
1
5125

110618
1
family

140106
1
genus

species
1

SRS097895\_contig\_number\_16345
979145

1
subclass
222545

37989
1
order

37990
family
1

genus
1
37991

species
1

SRS048620\_contig\_number\_contig-100\_7053.7054
2023133

phylum
1
5204

29000
1
subphylum

class
1
162481

1
order
231213

1799696
1
family

5533
genus
1

species
1

SRS023557\_contig\_number\_6375
1928479
